# *Chlorella vulgaris* Extract as a Serum Replacement That Enhances Mammalian Cell Growth and Protein Expression

**DOI:** 10.3389/fbioe.2020.564667

**Published:** 2020-09-15

**Authors:** Jian Yao Ng, Mei Ling Chua, Chi Zhang, Shiqi Hong, Yogesh Kumar, Rajeev Gokhale, Pui Lai Rachel Ee

**Affiliations:** ^1^Department of Pharmacy, Faculty of Science, National University of Singapore, Singapore, Singapore; ^2^Roquette Innovation Center, Helios, Singapore, Singapore; ^3^National University of Singapore (NUS) Graduate School for Integrative Sciences and Engineering, Singapore, Singapore

**Keywords:** *Chlorella vulgaris*, algae extract, serum replacement, 3D cell culture, CHO cells, bioprocess engineering, protein expression, tissue engineering and regenerative medicine

## Abstract

The global cell culture market is experiencing significant growth due to the rapid advancement in antibody-based and cell-based therapies. Both rely on the capacity of different living factories, namely prokaryotic and eukaryotic cells, plants or animals for reliable and mass production. The ability to improve production yield is of important concern. Among many strategies pursued, optimizing the complex nutritional requirements for cell growth and protein production has been frequently performed via culture media component titration and serum replacement. The addition of specific ingredients into culture media to modulate host cells’ metabolism has also recently been explored. In this study, we examined the use of extracted bioactive components of the microalgae *Chlorella vulgaris*, termed chlorella growth factor (CGF), as a cell culture additive for serum replacement and protein expression induction. We first established a chemical fingerprint of CGF using ultraviolet-visible spectroscopy and liquid chromatography-mass spectrometry and evaluated its ability to enhance cell proliferation in mammalian host cells. CGF successfully promoted the growth of Chinese hamster ovary (CHO) and mesenchymal stem cells (MSC), in both 2D and 3D cell cultures under reduced serum conditions for up to 21 days. In addition, CGF preserved cell functions as evident by an increase in protein expression in CHO cells and the maintenance of stem cell phenotype in MSC. Taken together, our results suggest that CGF is a viable culture media additive and growth matrix component, with wide ranging applications in biotechnology and tissue engineering.

## Introduction

Therapeutic proteins are an important class of medications for the treatment of complex diseases such as diabetes and immunological defects. The advent of recombinant DNA technology has enabled the production of these proteins in industrial-scale bioreactors. The process involves upstream genetic engineering of host systems, such as bacterial, yeast, insect, or transgenic plant and mammalian cells, along with downstream purification of extracted proteins (Tripathi and Shrivastava, 2019). In recent years, mammalian cells have become the main host systems for recombinant protein production (Walsh, 2014). This is due to their innate membranous organelles allowing post-translational modification (PTM) of the expressed proteins, which is critical for certain proteins to fold properly and/or exert its biological activity (Amann et al., 2019). In particular, given their ease of *in vitro* cell culture and ability to adapt to selection pressure, Chinese hamster ovary (CHO) cells are established as the workhorse for industrial protein production (Berger et al., 2020).

Commercial CHO cell lines (e.g., CHO-K1 and CHO-DG44) and their corresponding established transfection protocols have facilitated successful cell line development (Elshereef et al., 2019). However, the challenge of a cost-effective production lies in the costly and time-consuming upstream process development. Notably, tremendous research efforts are required for media composition optimization (Hunter et al., 2019). The conventional approach is focused on the development of an optimized cultivation media via component titration using the “one factor at a time” (OFAT) method (Ritacco et al., 2018). Recently, by understanding how certain intracellular anabolic pathways contribute to protein expression, there is considerable interest in improving protein production efficiency by tuning host cell metabolism via incorporation of expression pathway inducers into the cultivation media (Huang et al., 2017).

Substantial emphasis has also been placed on fetal bovine serum (FBS) replacement. The main components of FBS are proteins; and they include growth factors, hormones, and other functional proteins that are essential for cell growth and protein production (Gstraunthaler, 2003; Brunner et al., 2010). Despite the importance of FBS, several disadvantages in terms of batch-to-batch variations, risk of zoonosis, and high protein content complicating downstream purification process have motivated the search for serum-free medium formulations (van der Valk et al., 2018).

*Chlorella vulgaris* (*C. vulgaris*) is a unicellular microalgae discovered in 1890 by Martinus Willem Beijerinck (Krienitz et al., 2015). It is highly nutritious in nature and thus various forms of *C. vulgaris* extract are often consumed as a health supplement in some countries, notably Japan. Given *C. vulgaris*’ nutritious profile, some literatures have reported its potential use as an FBS alternative for *in vitro* cell culture. For example, Song et al. (2012) demonstrated that their hot water *C. vulgaris* extract stimulated proliferation of rat intestinal epithelial (IEC-6) cells after 24 h of incubation at serum-free conditions, via activation of the mitogen-activated protein kinase (MAPK), phosphoinositide-3-kinase–protein kinase B (PI3K/Akt), and canonical wingless-related integration site (Wnt) pathways. This study iterated that water extracts of *C. vulgaris* contained bioactive polysaccharides, that exerted important cell proliferative effects through gene induction pathways. These polysaccharides were later identified as phenolic compounds, which are usually found in algal or plant extracts bound to carbohydrates as glycosides (Safafar et al., 2015). *C. vulgaris* derived phenolic compounds possess potent antioxidative properties (Diaz-Vivancos et al., 2015), which may protect cells from serum deprivation-induced injury (Jung et al., 2017). Of interest, MAPK, PI3K/Akt and canonical Wnt are cross-species conserved signaling pathways that regulate protein expression (Proud, 2007), and manipulation thereof may potentially improve protein production yield (Chevallier et al., 2019). There is, however, to date no report that systematically evaluate the potential of *C. vulgaris* extract as a cell culture additive that could act as both a serum replacement and an inducer of protein expression.

In this report, we present the characterization of extracted bioactive components of *C. vulgaris*, termed chlorella growth factor (CGF), and demonstrate their ability to support cell growth in low serum conditions using high utility mammalian cell lines, such as CHO and mesenchymal stem cells (MSCs). The enhanced proliferative capacity in turn led to induced protein expression in CHO cells as measured by GFP protein marker expression. On the other hand, when used with MSCs, CGF stimulated their growth in both 2D and 3D cultures, while preserving their stem-like phenotype. Our results not only present an exciting opportunity for commercial protein production, but also for the development of novel MSC-based therapeutic strategies for tissue engineering and regenerative medicine (TERM).

## Materials and Methods

### Materials

Puromycin dihydrochloride (P8833), bovine serum albumin (BSA) lyophilized powder (A2153-10G), low-glucose (D5523), and high-glucose (D1152) Dulbecco’s Modified Eagle’s medium (DMEM), DMEM/Nutrient Mixture F-12 Ham, 1:1 mixture (D2906), Accutase solution (A6964), penicillin/streptomycin (P4333), paraformaldehyde (PFA, P6148), DAPI readymade solution (MBD0015-5ML), CelLytic^TM^ M (C2976-50ML), and Whatman^®^ 0.45 μm nylon membrane filters (WHA7404004) were purchased from Sigma-Aldrich (St Louis, MO, United States). All salts were received in anhydrous form. Fetal Bovine Serum (FBS) was obtained from Hyclone (South Logan, UT, United States). Ultrapure grade phosphate buffered saline (10× PBS) was procured from Vivantis Technologies Sdn Bhd (Subang Jaya, Selangor Darul Ehsan, Malaysia). Triton-X was obtained from Bio-Rad Laboratories (Hercules, CA, United States). CellTiter 96 AQueous One Solution Cell Proliferation Assay (MTS) was purchased from Promega (Madison, WI, United States). 0.22 μm polyethersulfone (PES) filters were acquired from sartorius stedim (Göttingen, Germany). Franz diffusion cells and Franz cell incubator, as well as HaCaT keratinocytes were kindly sponsored by Professor Paul Ho Chi Lui (NUS, Singapore). Human dermal fibroblasts (HDF), human Mesenchymal Stem Cells (hMSC, PT-2501) and Adipose-derived Stem Cells (ADSC, PT-5006) were purchased from Lonza Bioscience (Basel, Switzerland). Both GFP-transfected (puromycin) and native CHO-K1 cells were obtained from Accegen Biotech (Fairfield, NJ, United States). All other materials obtained are of research grade and used as received.

### CGF Extraction Method

Aqueous extraction of *C. vulgaris* biomass was performed at Roquette Singapore Innovation Center. *C. vulgaris* biomass harvest was first grinded into a powder form and suspended in deionized water to a concentration of 10% (w/v). The suspension was left to boil at 100°C for 20 min. After cooling down to room temperature, the suspension was centrifuged at 8,000 g for 30 min. Water-soluble bioactive components of *C. vulgaris*, termed as CGF, were harvested from the supernatant. This aqueous extract of CGF was sterile filtered through a 0.22 μm syringe filter prior to use for all studies.

### Characterization of CGF

#### Determination of Proximate Crude Composition of CGF

Direct protein quantification methods such as Bradford and Lowry assays are inaccurate for plant-based extracts (Biancarosa et al., 2017). Therefore, to determine the protein content of biomass extracts, a majority of studies employed an indirect nitrogen-protein (N-protein) conversion factor. By using HPLC (method described below) to measure N and Kjeldahl’s N-protein factor of 6.25, we determined the crude protein content of CGF and subsequently, calculated its carbohydrate content (Kjeldahl, 1883). Next, standardized methods of atomic absorption spectroscopy (AAS) and plate counting were used to determine the ash/trace metal and microbial contents of CGF respectively (Feldsine et al., 2002). The results were compared against prescribed limits for human use.

#### UV-Vis Spectroscopy

UV-Vis spectroscopy was used to quantify the amount of functional phenolic compounds in CGF. UV absorbance were measured at a wavelength of 260 nm (OD_260_) using Ultrospec 2100 pro UV-Vis spectrophotometer (Little Chalfont, United Kingdom). Briefly, 1 mL of diluted CGF samples were added into separate Starna quartz spectrophotometer cells (Ilford, United Kingdom) (*n* = 3). The solvent, either deionized water or 1× PBS, was used as blank. Linear calibration curves of blank corrected-OD_260_ against concentration of CGF were constructed for both solvents (Supplementary Figure S1). R^2^-values with weighted observation at 1/X^2^ for CGF concentration were computed to show a linear relationship between absorbance and the range of CGF concentration used.

#### LC-MS

The chemical composition of CGF was determined by high-performance liquid chromatography (HPLC) with mass selective detection, using Agilent 1200 series LC (Santa Clara, CA, United States) coupled with Bruker electrospray ionization (ESI) tandem mass spectrometer (micrOTOF-Q II) (Billerica, MA, United States). Components were separated using Merck Chromolith Performance RP-18 endcapped 100-3 HPLC column, 130 Å, 2 × 100 mm, 2.0 μm column (Kenilworth, NJ, United States), held at room temperature. Mobile phase consists of 1% (v/v) aqueous formic acid (A) and acetonitrile (ACN) (B), and was used with a discontinuous gradient; 0 min 5% B, to 40% B in 22 min, to 100% B in the next 15 min until the 62nd min, B decreased to 5% by the 63rd min, until the run ended on the 70th min, all at the flow rate of 0.2 mL/min. Chromatographic profiles were acquired in the wavelength at 260 nm. Injection volume was 20 μL (*n* = 3). Eluted components were ionized by ESI source, using N_2_ for nebulization (pressure of 2.0 bar) and drying (flow of 6 L/min, temperature of 200°C). Set capillary voltage was 3,500 V, end plate offset −500°V, collision cell RF 250.0 Vpp. Data were acquired in MS (auto) scanning mode between 50 to 2,000 mass-to-charge ratio (m/z). The entire LCMS experiment was repeated for three separate batches of CGF (*n* = 3), statistical comparison of characteristics peak areas was then performed with one-way ANOVA using GraphPad Prism v8.4.3.

### Cell Culture

#### *In vitro* Expansion of Mammalian Cell Lines

All mammalian cells received in cryovials were thawed and cultured on Nunc T-75 cell culture flasks (Roskilde, Denmark), according to the manufacturer’s protocol. HDF and HaCaT cells were expanded using high-glucose DMEM, while hMSC and ADSC were expanded using low-glucose DMEM. GFP-transfected and native CHO-K1 cells were expanded in DMEM/Nutrient Mixture F-12 Ham 1:1 mixture media, with or without 20 mg/mL of puromycin (selection pressure) respectively. All culture media were supplemented with 10% (v/v) FBS and 1% (v/v) penicillin-streptomycin. HDF, HaCaT, and CHO-K1 cells were used between passage 3–20. The stem cells were used between passages 3–5. All cells were harvested between 80 and 90% confluency.

#### Cell Encapsulation Into Hydrogel Matrix for *in vitro* 3D Cell Culture

A cell-adhesive gellan gum-collagen hydrogel scaffold was used for *in vitro* 3D cell culture. The hydrogel matrix was prepared as previously reported (Ng et al., 2019). Briefly, 60 μL of cell-laden hydrogels were formed in each well of 96-well plate (HDF) or 48-well plate (hMSC or ADSC), and supplemented with 200 and 600 μL of culture media respectively. All cultured cells were incubated at 37°C in a humidified atmosphere of 5% CO_2_ in air. The culture media were replaced every 2–3 days (Mon, Wed and Fri).

### Cytotoxicity Assay

The effect of CGF on cell viability was examined using the colorimetric MTS assay, according to the manufacturer’s protocol. Mammalian cells were harvested using Accutase solution and seeded at 5,000 cells per well of 96-well plates (*n* = 3 per concentration of CGF). The plates were incubated for 24 h to allow cell adhesion. After which, the spent media were replaced with fresh media containing varying final concentrations (w/v) of CGF and further incubated for 72 h. Next, 10 μL of MTS was incubated with the cells per 100 μL of culture media for another 3 h to allow the conversion of MTS into a brown formazan product. Then, 100 μL of the supernatant of each well was transferred to a new well in a 96-well plate. The measurement of total metabolic activity was done on Hidex Sense microplate reader (Turku, Finland) at OD_490_.

Cell-free wells were used as blank controls, untreated cells were used as negative controls. Relative cell viability was determined when blank-corrected OD_490_ values of cells exposed to different concentrations of CGF were expressed against blank-corrected OD_490_ of negative controls. The concentration of CGF that induced highest cell viability (E_max_) and killed 50% of the cell population (CC_50_) were tabulated. For 3D cell culture, cell-free hydrogels were used as blank controls and untreated encapsulated HDF were used as negative controls instead.

### Serum Replacement Assay

At their respective E_max_ concentrations, the effect of CGF on proliferation of mammalian cells cultured in serum-reduced conditions was examined using the colorimetric MTS assay as described above. Cells were seeded in either serum-free (0% FBS), low serum (2% for HDF, HaCaT and CHO-K1, or 5% for hMSC and ADSC) or full serum (10% FBS) culture media [*n* = 3 (2D cell culture) and = 9 (3D cell culture) per concentration of serum, per time point]. MTS assays were performed on day 0 (6 h after seeding) to determine the cells’ initial viability, and on days 1, 3, and 7 of incubation, as well as days 14 and 21 for encapsulated stem cells. Cell proliferation was determined by expressing the blank-corrected OD_490_ values obtained from each time points against that of day 0’s.

### Flow Cytometry Analysis

After 21 days of incubation with CGF at serum-reduced conditions, multipotency of ADSC was assessed via flow cytometry analysis of cell surface protein markers, using the CytoFLEX LX flow cytometer (Beckman Coulter, CA, United States). Briefly, ADSC was first harvested using Accutase solution and resuspended in 0.1% BSA in 1× PBS at the cell density of 1 × 10^6^ cells/mL. The cells were then stained with FITC anti-human CD105 (Clone MEM-229), CD73 (Clone AD2), and CD90 (Clone 5E10), as well as PE/Cy5.5 anti-human CD45 (all Abcam, Cambridge, United Kingdom), at 4°C for 30 min in the dark, according to the manufacturer’s protocol. Untreated and unstained ADSC was assessed in the same manner for the quadrants to be drawn. Percentage of positive cells for each marker was calculated from three independent experiments (*n* = 3).

### Protein Expression via GFP-Transfected CHO Cell Line

GFP-transfected or native CHO-K1 cells were seeded separately with 1 mL of 10% FBS puromycin-free culture media at the cell density of 5 × 10^4^ cells/mL, in each Ibidi 35 mm glass bottom dish (*n* = 3 per group) (Martinsried, Germany), overnight for the cells to attach. After which, the cells were first rinsed with sterile 1× PBS, then exposed to 0% FBS (serum-free) puromycin-free culture media supplemented either with or without 0.001 g/L of CGF for 72 h. The four groups were labeled as GFP-transfected CHO-K1 “GFP (+)” or native CHO-K1 cells “GFP (−),”supplemented with “CGF (+)” or without “CGF (−)” CGF.

#### Enzyme-Linked Immunosorbent Assay (ELISA)

Quantification of intracellular GFP was conducted with Abcam GFP SimpleStep ELISA kit (ab171581, Cambridge, United Kingdom) on cell lysates that were harvested using CelLytic M, according to the manufacturer’s protocol. Using GFP standards provided in the kit and Tecan Infinite M Plex plate reader (Männedorf, Switzerland), a calibration curve of blank-corrected absorbance intensity against the concentration range of GFP was drawn. The concentration of GFP from the four different experimental groups were calculated from the calibration curve.

#### Confocal Microscopy

Green fluorescence emitted from GFP was observed with confocal laser scanning microscopy (CLSM). Briefly, cells were fixed with 1 mL of 4% PFA for 10 min, and subsequently permeabilized with 1 mL of 0.1% triton-x for 3 min. After which, cells’ nuclei were counter-stained with 1,000× -diluted DAPI readymade solution for 20 min. Cell fixation, permeabilization and staining were all conducted at room temperature in dark. Confocal images were captured with Zeiss LSM710 (Oberkochen, Germany) using the 20×/0.8 objectives, with 405 nm diode and 488 nm argon laser as the excitation sources. Images were processed and exported using ZEN lite 2.3 (Carl Zeiss). The GFP intensity of each sample (*n* = 3 per group) was normalized against its own DAPI intensity using the ImageJ software. The average normalized GFP intensities of all groups were then statistically analyzed and compared.

### Statistical Analysis

Statistical analysis was performed using GraphPad Prism v7.0.3. Data are presented as mean ± SD. One-way ANOVA was calculated between different experimental groups, with subsequent Bonferroni *post-hoc* tests performed to determine statistically significant differences (^∗^*p* < 0.05, ^∗∗^*p* < 0.005, ^∗∗∗^*p* < 0.0005, ^****^p < 0.0001) against controls.

## Results and Discussion

### Chemical Fingerprint of CGF

**FIGURE 1:**
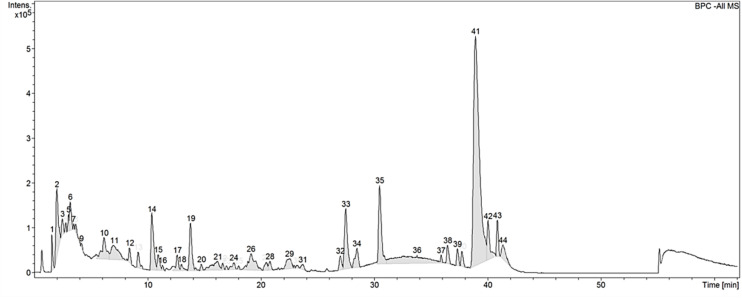
Base peak chromatogram (BPC) of CGF analyzed with HPLC on Agilent 1200 series LC. Mobile phase consists of 1% (v/v) aqueous formic acid and acetonitrile (ACN) and, they were used with a discontinuous gradient. Flow rate was 0.2 mL/min. UV detection was set at the wavelength of 260 nm and, 20 μL of 10% (w/v) CGF was injected each run. The ordinate and abscissa are UV absorbance intensity (mAU) and retention time (*t*_R_) respectively.

Table 1 shows the proximate crude composition of CGF. The soluble extract contained approximately 67.1% crude protein, 27.4% carbohydrate, and 5.7% crude ash, which is in good agreement with a previous study (Song et al., 2012). In addition, the trace metals and microbial contents of CGF were found to be within the prescribed limits according to Singapore Medicines Act for human usage (Koh and Woo, 2000). Next, to establish the chemical fingerprint of CGF, phenolic compounds within CGF were separated by liquid chromatography, and the precursor ions present in each peak were subjected to collision-induced associated (CID) mass spectrometry analysis. The base peak chromatograms (BPC) for three separate batches of CGF are shown in Figure 1. The representative retention time (*t*_R_) and area under curve (AUC) of each peak of CGF are presented in Supplementary Table S1. A total of 44 peaks were identified in the chromatogram and the major phenolic component of CGF was eluted at the 41st min. Characteristics peak areas were compared between the chromatograms of three separate batches of CGF (Supplementary Figure S2) and were not found to be statistically different (*P* = 0.9885). Our results indicate that our method of extracting CGF from different *C. vulgaris* harvests have minimal batch-to-batch variation.

**TABLE 1:** Proximate crude composition of *C. vulgaris* extract, CGF.

Moisture (%)	Crude protein^†^ (%)	Crude carbohydrate^‡^ (%)	Crude ash (%)	Trace metals^§^ (mg/kg)	Microbial content^¶^ (CFU/g)
**Chlorella growth factor (CGF)**					
5.5	67.1	27.4	5.7	Arsenic < 0.20	Total aerobic microbe < 110
				Mercury < 0.02	*E. Coli* undetectable
				Cadmium < 0.10	Salmonella species undetectable
				Lead < 0.20	

*^†^Crude protein composition = expressed as percentage of dry base. ^‡^% carbohydrate = 100% - % moisture - % protein - % lipid (assumed to be 0% in aqueous extract of C. vulgaris). ^§^Trace element content complied with Singapore Medicines Act, under Medicines (Prohibition of Sale and Supply) Order for prohibition of sales or supply of medicinal products containing excessive levels of toxic heavy metals -p3 (1) (c). ^¶^Microbial content complied with Singapore Medicines Act, under Medicines (Prohibition of Sale and Supply) Order for prohibition of import, sale or supply of Chinese proprietary medicine containing excessive microbial contamination -p4.*

Preliminary full scan mode yielded mass spectra with signal between 50 and 2,000 m/z, hence for increased sensitivity, subsequent scans were limited to this mass range. Using Bruker’s SmartFormula structure elucidation algorithm, the most probable molecular formulae of 39 precursor ions were deduced. The major phenolic component of CGF has the molecular formula of C_16_H_31_O_2_. For all proposed molecular formulae, their molecular weights were concluded on the basis of their negative ion ESI mass spectra, which showed precursor ions as [M – H]^–^. A full list of possible molecular formulae as well as their calculated exact masses are presented in Supplementary Table S1. For accurate mass reporting, the tolerance for maximum mass deviations (measurement errors) of identified precursor ions were kept to less than ±0.001 Da. Standardization of aqueous extractions of *C. vulgaris* to obtain CGF are based on identical BPC and mass spectra for all our subsequent studies.

### CGF Promoted Cell Proliferation at Serum-Reduced Conditions in Mammalian Cell Types of Industry Significance

#### Cytotoxicity Assay

**FIGURE 2:**
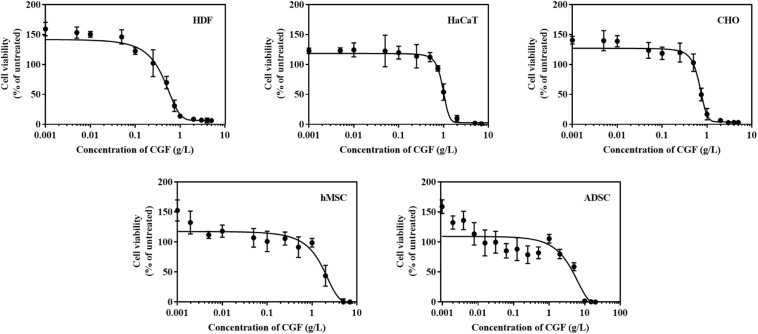
Effect of various concentrations of CGF on the cell viability of HDF, HaCaT, CHO, hMSC, and ADSC (2D cell culture). Cells were cultured on 96-well plates with increasing concentrations of CGF for 72 h. Their cell viability was determined via MTS assay. For each concentration of CGF, the cell viability was expressed against untreated cells to obtain a percentage cell viability value. The resultant percentages of cell viability were plotted against concentrations of CGF. E_max_ and CC_50_ values of CGF for each cell lines were interpolated from the graphs. The mean (*n* = 3) ± SD are shown.

*C. vulgaris* extracts have been shown to induce cell proliferation in cell lines such as IEC-6, HepG2, and MOLT-4 cells (Saad et al., 2006; An et al., 2010; Yusof et al., 2010). These evaluations are however limited in cells directly relevant to the medical and biotech industry. To address this gap, we thus selected 5 mammalian cell lines of industry significance and performed cytotoxic assays over a range of CGF concentrations (Figure 2). At low concentrations, CGF induced cell proliferation with a maximum increase in cell viabilities at E_max_ of 0.001 g/L for all cell types. This is in good agreement with previous studies which have shown that extracts of *C. vulgaris* are cell proliferative at lower concentrations while being cytotoxic at high concentrations (Yusof et al., 2010; Song et al., 2012). In addition, our study revealed that variable cytotoxicity was observed amongst mammalian cell lines, with HDF displaying the highest sensitivity with an average CC_50_ value of 0.597 g/L (95% CI: 0.508–0.710) and ADSC displaying the lowest sensitivity at CC_50_ value of 4.645 g/L (95% CI: 2.932–7.172) (Supplementary Table S2).

#### Serum Replacement Assay

**FIGURE 3:**
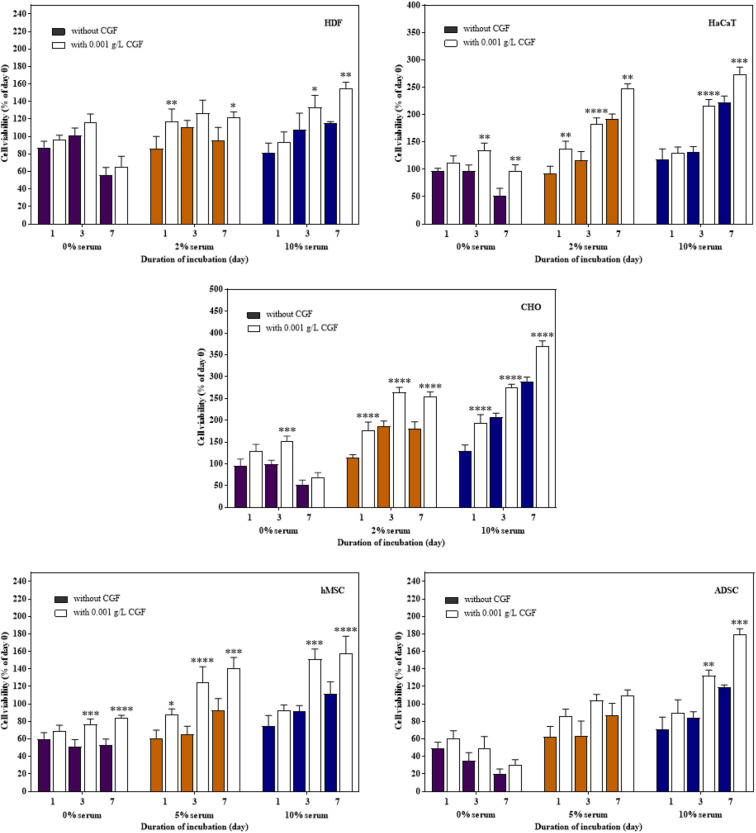
Effect of CGF at its E_max_ concentration on the cell viability of HDF, HaCaT, CHO, hMSC, and ADSC at varying serum concentrations (2D cell culture). Cells were cultured on 96-well plates with no serum (0%), low serum (2% for HDF, HaCaT, CHO, and 5% for hMSC and ADSC), or full serum (10%) culture media, supplemented with or without CGF, for 7 days. Cell viability was determined at referred time points via MTS assay. The cell viability was then expressed against cells seeded for 6 h on day 0 to obtain a percentage cell viability value. The resultant percentages of cell viability were plotted against duration of incubation. The mean (*n* = 3) ± SD are shown. Statistical comparisons were made between cells incubated with CGF against cells incubated without CGF of the same duration of incubation (^∗^*p* < 0.05, ^∗∗^*p* < 0.005, ^∗∗∗^*p* < 0.0005, ^*⁣*⁣**^p < 0.0001).

Therapeutic proteins are produced through cell fermentation in a culture media that generally consists of growth factors derived from serum of an animal origin. To avoid complications associated with animal blood-derived ingredients, animal component-free chemically defined media are currently gold-standard alternatives (Schnellbaecher et al., 2019). However, these cell-line and cell-clone specific formulations often require extensive optimization before the ideal composition could be identified (Lakshmanan et al., 2019; Marigliani et al., 2019). In another approach, from the classic example of Advate and Recombinate, cell lines could also be adapted to serum-free culture (Négrier et al., 2008). Unfortunately, this method can only be applied to a very limited number of cell types without deleterious phenotypic alterations (van der Valk et al., 2018). To investigate the role of CGF as an alternative cell growth additive, we herein subjected mammalian cells to growth supplementation with CGF at full serum and serum-reduced conditions. As shown in Figure 3, significant increase in cell proliferation with CGF supplementation at E_max_ of 0.001 g/L was observed for all cell types at both conditions as compared to those cultured without CGF. Specifically, after 7 days of culture, mammalian cells exposed to CGF in low serum conditions exhibited between 30 and 70% increase in cell viabilities. Given that stem cells are notoriously sensitive to culture media composition, we made significant observations when their cell viabilities recovered from 85 to 140% upon CGF treatment at serum reduced conditions. However, in the complete absence of serum, CGF displayed only limited effect in reversing cell death over a period of 7 days. Taken together, our results suggest that CGF-enriched media enables a wide range of previously non-adapted high-utility mammalian cells, such as CHO and stem cells, to grow in serum-reduced conditions without disadvantages associated with conventional strategies.

### CGF Supplementation Preserved Mammalian Cell Function and Phenotype

**FIGURE 4:**
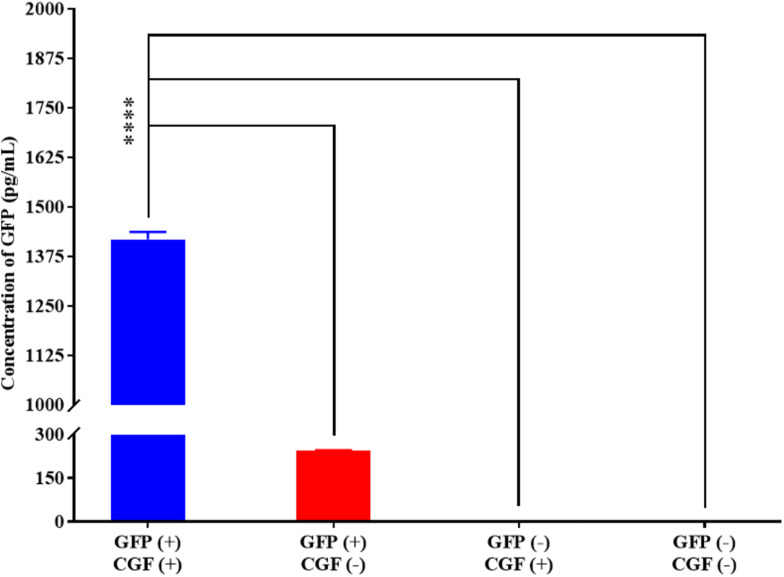
Quantification of GFP expressed by CHO-K1 cells upon exposure to CGF as determined from ELISA. Significantly higher concentration (∼5.60-fold) of GFP could be detected in GFP-transfected CHO cells treated with CGF “GFP (+), CGF (+)” as compared to GFP-transfected CHO cells not treated with CGF “GFP (+), CGF (−)” as well as native CHO cells treated with CGF “GFP (−), CGF (+)” or without CGF “GFP (−), CGF (−)” (^∗^*p* < 0.05, ^∗∗^*p* < 0.005, ^∗∗∗^*p* < 0.0005, ^*⁣*⁣**^p < 0.0001).

**FIGURE 5:**
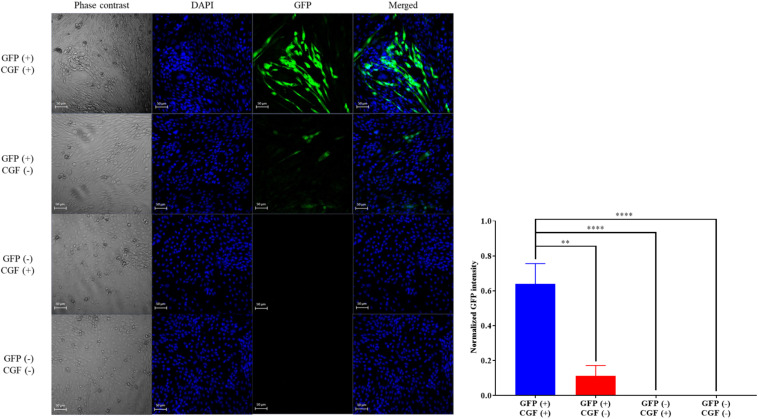
Confocal images of GFP-transfected and native CHO cells with or without exposure to CGF. Visualization of intracellular GFP fluorescence via CLSM was performed at 20× zoom. Cells’ nuclei were counter-stained with DAPI. Phase contrast images were taken for reference to cell boundaries. Images of GFP and DAPI were merged for co-localization purposes. Visually, the GFP fluorescence intensities are significantly higher in GFP-transfected CHO cells treated with CGF “GFP (+), CGF (+)” as compared to GFP-transfected CHO cells not treated with CGF “GFP (+), CGF (−)” as well as native CHO cells treated with CGF “GFP (−), CGF (+)” or without CGF “GFP (−), CGF (−)”. Normalized GFP intensity of GFP (+), CGF (+) CHO cells were statistically significantly higher than the other groups (^∗^*p* < 0.05, ^∗∗^*p* < 0.005, ^∗∗∗^*p* < 0.0005, ^*⁣*⁣**^p < 0.0001).

To show that CGF not only enhance cell growth but also preserve their function, we quantified GFP production using the stably transfected GFP reporter-producing cell line, CHO-K1, in the absence and presence of CGF (Lo et al., 2015). GFP is constitutively expressed in the presence of 20 mg/mL of puromycin in CHO-K1 but intracellular GFP degrades rapidly when protein synthesis is inhibited (Halter et al., 2007). As shown in Figure 4 and Supplementary Figure S3, at serum-free conditions, CGF supplementation resulted in ∼5.6-fold increase in GFP expression as compared to that without CGF treatment. As expected, GFP could not be detected from the cell lysates of wild-type CHO-K1 cells with or without CGF supplementation, indicating that increased GFP expression was a result of activation of protein expression pathways by CGF in transfected CHO cells. Confocal images supported our observations and showed visually that the fluorescence intensities correlated with CGF supplementation in the cells (Figure 5). Similarly, normalized GFP intensity of the confocal images showed ∼5.7-fold increase when GFP CHO cells were supplemented with CGF. Hence. our data presents an exciting opportunity for bioprocess re-engineering via CGF supplementation for enhanced commercial protein production.

**FIGURE 6:**
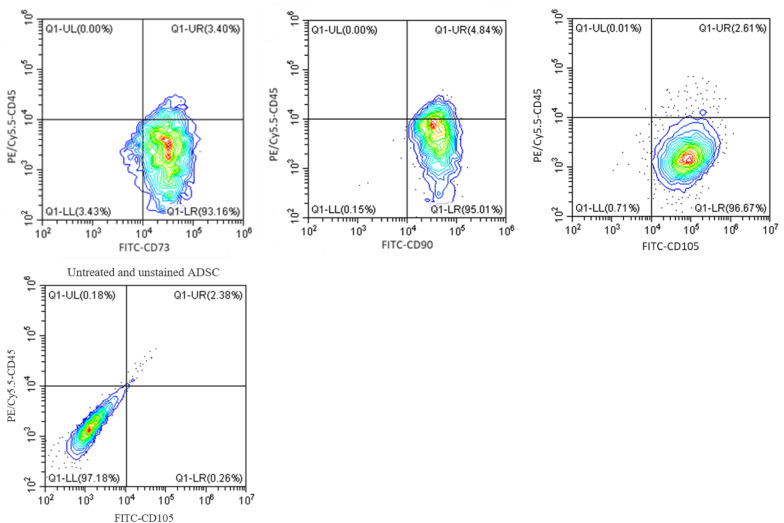
Flow cytometry analysis of ADSC exposed to CGF for 21 days. The analysis was performed with gates set on CD73/90/105 + CD45- cell population. Representative flow cytometry contour-plots showed that a majority of the cell population (>95%) exhibited canonical CD73, CD90, and CD105 mesenchymal stem cell surface markers. Whereas, only a small population (<5%) of cell population was positive for the hemopoietic stem cell surface marker, CD45. This indicated that multipotency of ADSC was retained after culture with CGF. A contour plot of unstained ADSC below denotes how the quadrants were drawn. The data represent analyzed cultures in triplicates.

Next, to confirm that CGF does not affect the maintenance of cell phenotype with continuous expansion, we performed flow cytometry using specific antibodies against canonical cell surface markers in ADSC through five passages. Figure 6 shows that ADSC maintained a stem-like phenotype with multiple passages under CGF supplementation. The majority of ADSC (>95%) was shown to express MSC markers CD73, CD 90 and CD105, while a negligible population (<5%) expressed the hematopoietic marker CD45. This gives confidence to the use of CGF in cell expansion designated for TERM applications. Given prior studies have shown that plant extracts are capable of stimulating differentiation in MSC, it is important to conduct future complementary investigations to understand the effect of CGF on the differentiation potential of 3D-cultuired ADSC (Zhang et al., 2015; Imran et al., 2017; Saud et al., 2019).

### CGF Supports 3D Cell Culture for TERM Applications

**FIGURE 7:**
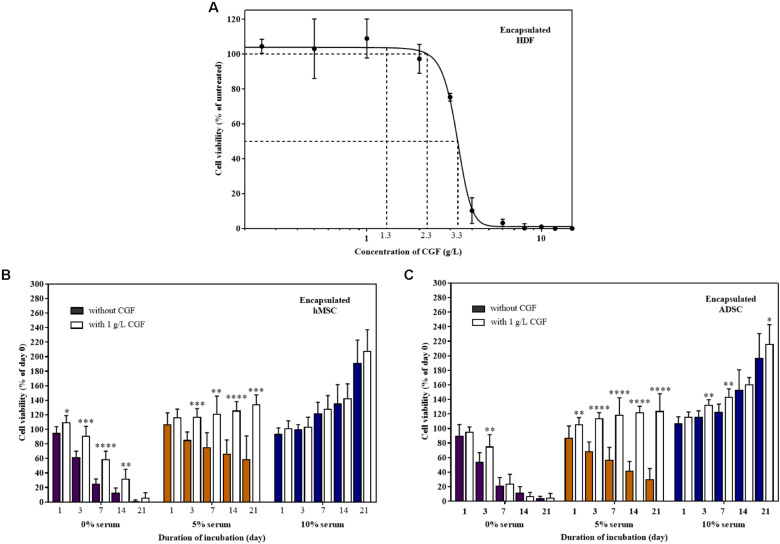
Effect of CGF on HDF, hMSC, and ADSC cultured in 3D hydrogel scaffolds. **(A)** Effect of various concentrations of CGF on the cell viability of encapsulated HDF. Cells were encapsulated and cultured on 48-well plates with increasing concentrations of CGF for 72 h. Their cell viability was determined via MTS assay. For each concentration of CGF, the cell viability was expressed against untreated cells to obtain a percentage cell viability value. The resultant percentages of cell viability were plotted against concentrations of CGF. The mean (*n* = 3) ± SD are shown. Effect of CGF at its E_max_ concentration on the cell viability of encapsulated **(B)** hMSC and **(C)** ADSC. Cells were encapsulated and cultured on 48-well plates with no serum (0%), low serum (5%), or full serum (10%) culture media, supplemented with or without CGF, for 21 days. The cell viability was then expressed against cells seeded for 6 h on day 0 to obtain a percentage cell viability value. The resultant percentages of cell viability were plotted against the duration of incubation. The mean (*n* = 9) ± SD are shown. Statistical comparisons were made between cells incubated with CGF against cells incubated without CGF of the same duration of incubation (^∗^*p* < 0.05, ^∗∗^*p* < 0.005, ^∗∗∗^*p* < 0.0005, ^*⁣*⁣**^p < 0.0001).

TERM is concerned with the replacement of damaged tissues with *in vitro* created tissues (Gomes et al., 2017; Liu et al., 2017). For the artificial construction of living tissues, the tri-component recipe of a tissue scaffold, stem cells, and animal-derived growth factors are quintessential (Ng et al., 2020). To replicate 3D cell culture, MSCs encapsulated in a previously reported hybrid gellan gum-collagen hydrogel (Ng et al., 2019) were grown with and without CGF in both full and reduced serum conditions. The hydrogels exhibited consistent high degree of porosity (Supplementary Figure S4) with a maximal pore diameter of 150 μm and did not impede the effective movement of relatively smaller sized bioactive components of CGF (Supplementary Table S3) in the matrix to reach the encapsulated mammalian cells according to the Franz cell diffusion assay (Supplementary Figure S5). First, we determined the E_max_ and CC_50_ of CGF to be 1 and 3.314 g/L (95% CI: 3.150–3.489 g/L) respectively, using hydrogel encapsulated HDF (Figure 7A). Next, at this E_max_ concentration, CGF exerted the most significant effect at reduced serum conditions (5%) by increasing stem cell viabilities beyond 120% after 3 weeks of culture (Figures 7B,C). This is a 2- to 4-fold increase in enhancement as compared to cells grown in the absence of CGF.

However, similar to 2D cell culture, in the complete absence of serum, CGF could not reverse cell death, although it could potentially delay the process as shown with encapsulated MSCs. Expectedly, CGF did not make any difference in full serum conditions, given the supraphysiological levels of growth factors present. Nonetheless, partial serum replacement with the use of algal-derived CGF as a growth matrix component in TERM may in part circumvent pertinent biosafety concerns associated with animal-derived serum for artificial tissue implantation such as xeno-immunization and zoonosis.

## Conclusion

We have shown that CGF, an aqueous extract of *C. vulgaris*, was able to enhance cell proliferation in mammalian cell lines of biomedical significance in both *in vitro* 2D and 3D cell culture, with the subsequent increase in protein production yield and maintenance of stem cell phenotype. This highlights the exciting use of CGF as an industry-relevant biocatalyst for increasing therapeutic protein production yield, as well as a cell growth component for TERM applications.

## Data Availability Statement

All datasets generated for this study are included in the article/Supplementary Material.

## Author Contributions

JN, RG, and PE conceived the direction of the research work. CZ and SH extracted *C. vulgaris* into CGF. MC conducted the 2D cell culture studies. JN conducted the other experiments. JN and PE analyzed the data and wrote the manuscript. CZ, SH, and YK reviewed and edited the manuscript. RG and PE provided technical guidance and edited the manuscript drafts. All authors contributed to the article and approved the submitted version.

## Conflict of Interest

CZ, SH, YK, and RG were employed by the company Roquette Asia Pacific Pte Ltd., Singapore. The remaining authors declare that the research was conducted in the absence of any commercial or financial relationships that could be construed as a potential conflict of interest.
