# Supplementary material for: Chlorella vulgaris Extract as a Serum Replacement That Enhances Mammalian Cell Growth and Protein Expression
Source: Front Bioeng Biotechnol. 2020 Sep 15;8:564667. doi: 10.3389/fbioe.2020.564667 (PMC7522799; doi:10.3389/fbioe.2020.564667)
Supplement: Supplementary file 1 [file Data_Sheet_1.docx]

Supplementary Material


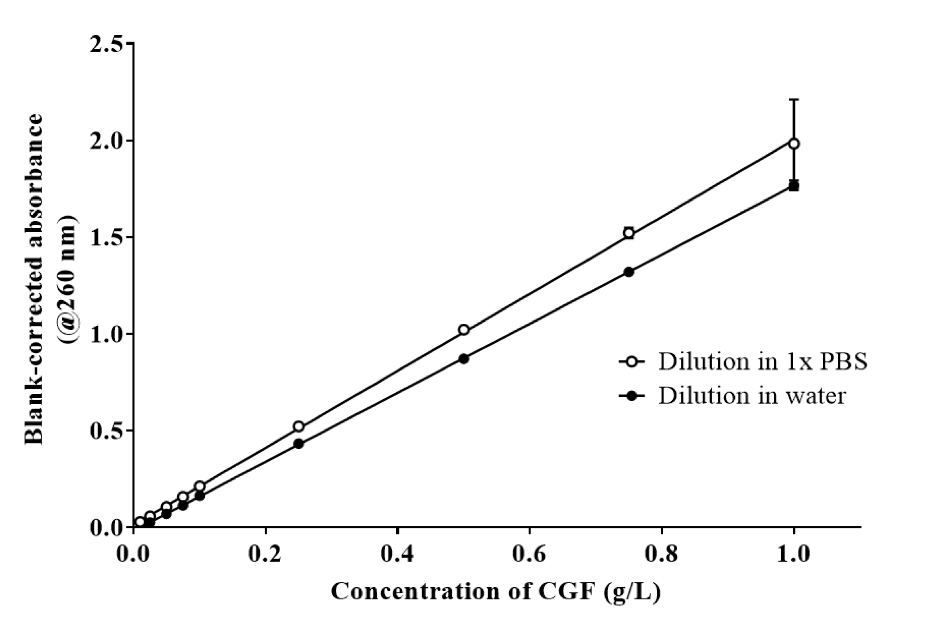
**Figure S1**. Calibration curve of blank-corrected absorbance intensity against various concentrations of CGF diluted in water or 1x PBS. Blank absorbance values were obtained when the solvent, either deionized water or PBS, was examined. The mean (n = 3) ± SD are shown. The linear equations are Y = [(1.785 × X) - 0.01677] and Y = [(1.99 × X) + 0.01468] when CGF is diluted in water and PBS respectively. Y refers to the absorbance intensity at 260 nm (OD_260_) while X refers to the concentration of CGF in g/L. Weighted R^2^ values are 0.9928 and 0.999 for PBS and water respectively. Unknown concentration of CGF, diluted in PBS, can be calculated from the formula: [CGF]g/L = [(abs_260_ - 0.01468)/1.99].

**Figure S2**. Base peak chromatograms (BPC) of three batches of CGF analyzed with HPLC on Agilent 1200 series LC. Mobile phase consists of 1% (v/v) aqueous formic acid (A) and acetonitrile (ACN) (B) and, they were used with a discontinuous gradient. Flow rate was 0.2 mL/min. UV detection was set at the wavelength of 260 nm and, 20 μL of 10% (w/v) CGF was injected each run. The ordinate and abscissa are UV absorbance intensity (mAU) and retention time (*t*_R_) respectively. Peak patterns appear to be similar between the three BPCs.


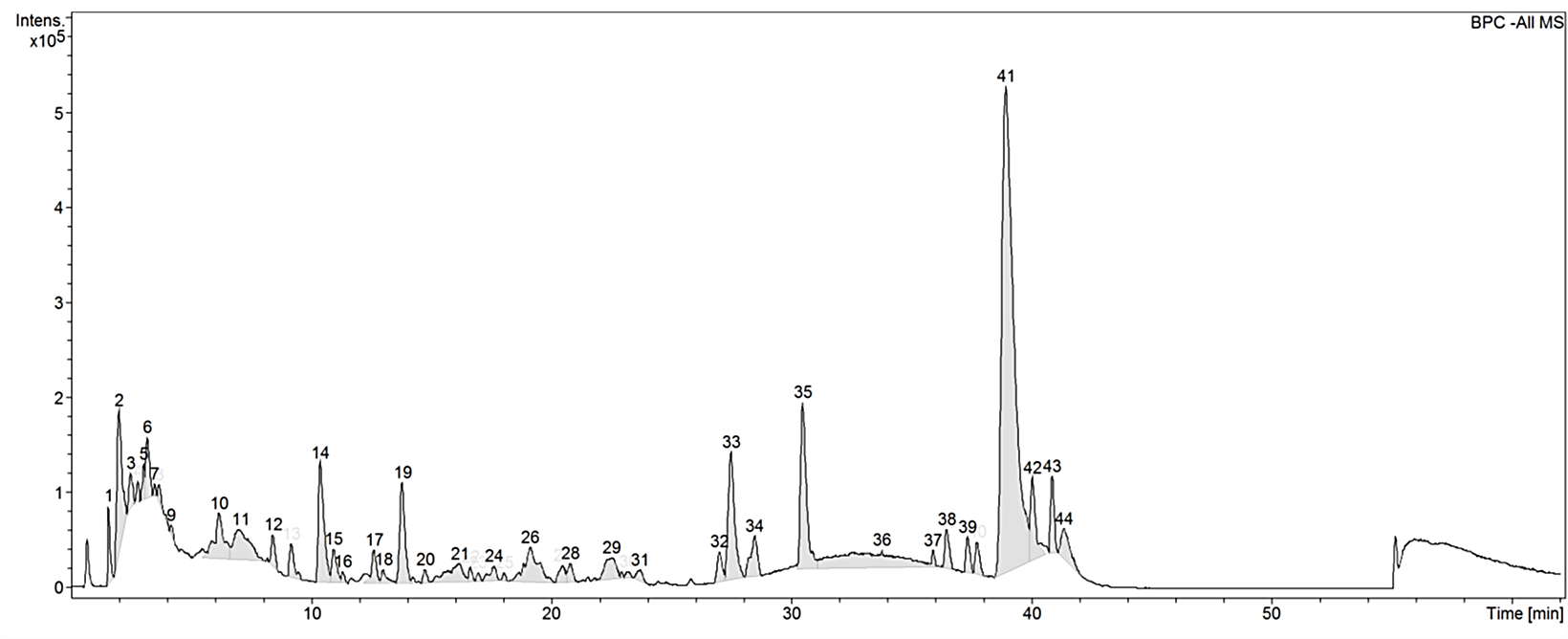


Batch 1


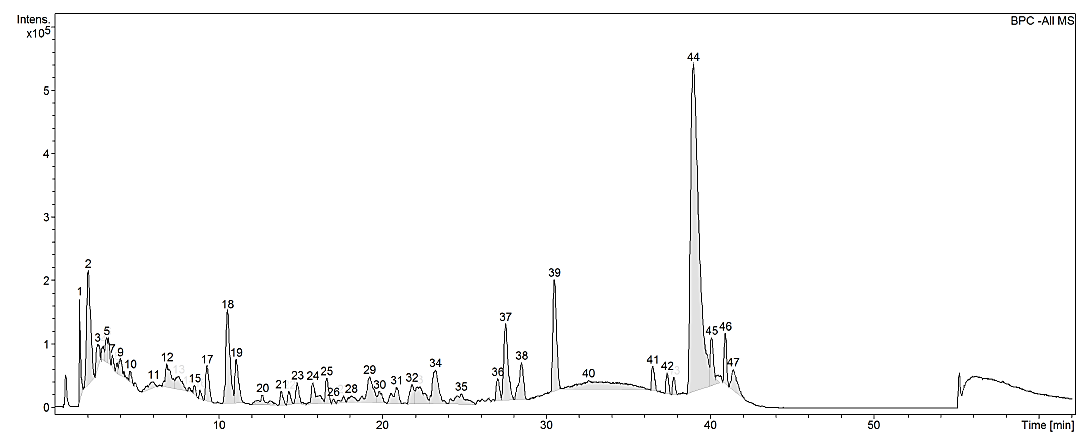


Batch 2


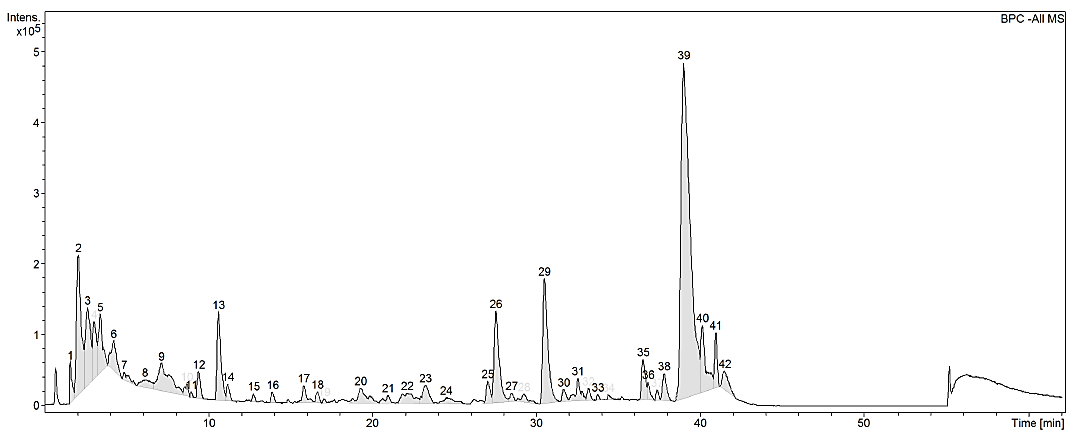


Batch 3


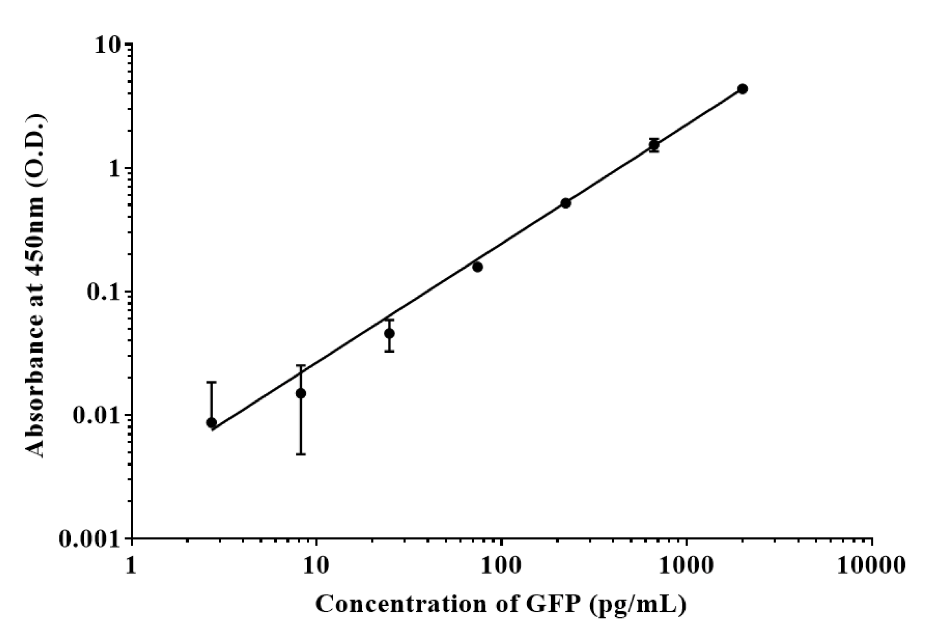
**Figure S3**. Calibration curve of blank-corrected absorbance intensity against various concentrations of GFP standards. Blank absorbance values were obtained when the kit’s diluent was examined. The mean (n = 3) ± SD are shown. The linear equation is Y = [(0.9632 × X) – 2.538]. Y refers to the absorbance intensity at 450 nm (OD_450_) while X refers to the concentration of GFP in pg/mL. R^2^ value is 0.9946. Unknown concentration of GFP can be calculated from the formula: [GFP]pg/mL = [(abs_450_ + 2.538)/0.9632].

***Hydrogel matrix porosity via scanning electron microscopy (SEM)***

The microstructure and porosity of the hydrogel matrix were analysed using SEM. In brief, lyophilized hydrogel samples (Alpha 1-2 LD+, Christ Martin, Germany) were first frozen in liquid nitrogen before being separated at their cross-sections. Then, the dissected hydrogels were quickly mounted onto pin stubs with their cross sections facing the electron emission source. The samples were subsequently sputter coated with gold using the JEOL JFC-1100 ion sputter (Tokyo, Japan) and examined with JEOL JSM-6510 scanning electron microscope. Pore size was calculated with reference to an in-built scale bar.


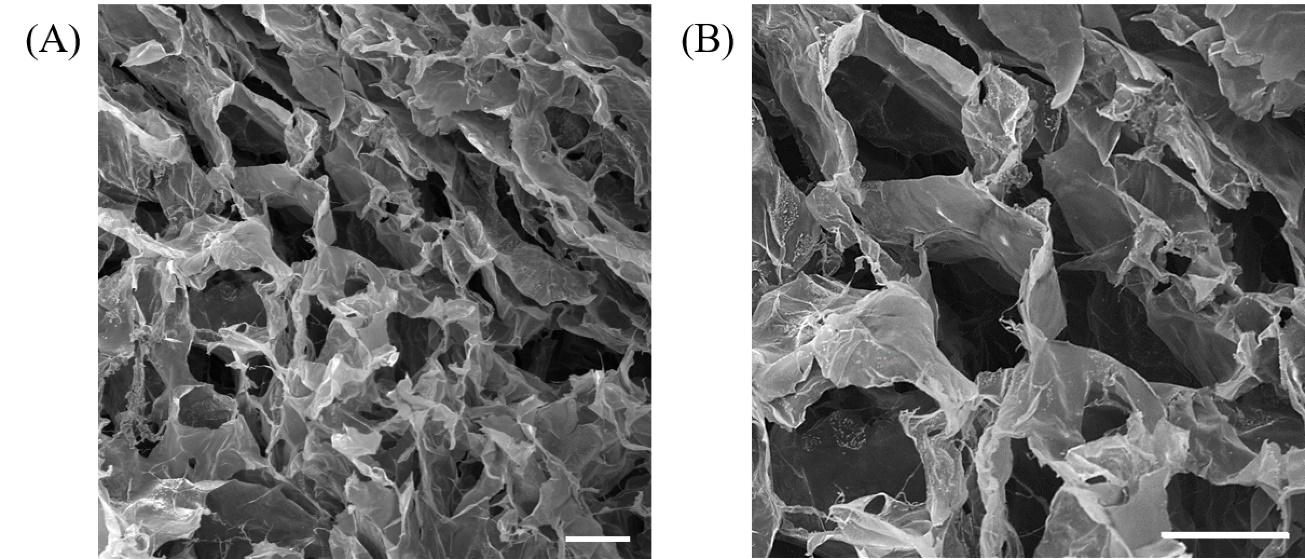
**Figure S4**. Scanning electron microscopy (SEM) images of the cross-section of hydrogel scaffold used for 3D cell culture at (A) 150× and (B) 250× zoom. Hydrogel samples were lyophilized to preserve their microstructure. Under the SEM, regularly shaped pores are clearly visible throughout the field of view. The longest diameter of the pores is defined as the further perpendicular distance between 2 points of the pores’ circumference, and was estimated to be ~ 150 µm. Scale bar = 100 µm.

**Franz diffusion cell assay**

Vertical Franz diffusion cells with an effective area of 1 cm^2^ were used to determine the diffusion of CGF across the hydrogel matrix (Franz, 1975). The device is divided into the donor and receptor compartments, which were separated by two sheets of Whatman^®^ 0.45 µm nylon membrane filters. 240 µL of cell-free hydrogel was formed in the donor compartment of Franz diffusion cells (n = 3), each donor compartments were then topped up with 800 µL of 1 g/L of CGF diluted in 1x PBS. The receptor compartment contained 5 mL of 1x PBS and a small magnetic stirrer. The Franz cells were sealed with parafilm to minimize the effect of evaporation and clamped to secure the overall setup. The assembled Franz cells were then placed inside a hot air chamber (Franz cell incubator) with the temperature maintained at 37 °C. Magnetic stirrers in the receptor cells stirred at a constant speed of 180 rpm. CGF solution in the donor compartments was replaced every 2 – 3 days (Mon, Wed and Fri).

For quantification, samples were removed from the receptor compartment via the sampling port at different time-points after 1, 3, and 7 days of incubation. Fresh 1 mL 1x PBS was immediately refilled after the sampling was completed. The concentration of CGF (g/L) released at each time-point (C_a_) was determined by UV spectroscopy at OD_260_. The total weight of CGF (g) within the receptor compartment was then quantified. The amount of CGF (g) removed from the previous time points (W_o_) was added to determine the total amount of CGF collected at each referred time point. The resultant value was then expressed against the total amount of CGF (g) added to the donor compartment (W_t_). Cumulative percentage release was then determined based on equation (1):

$$Cumulative \% release of CGF =\left( \frac{\left\{ \left[ \frac{Ca}{1000} \times5 \right]+Wo \right\}}{Wt} \right)\times100\%-\left( 1 \right)$$

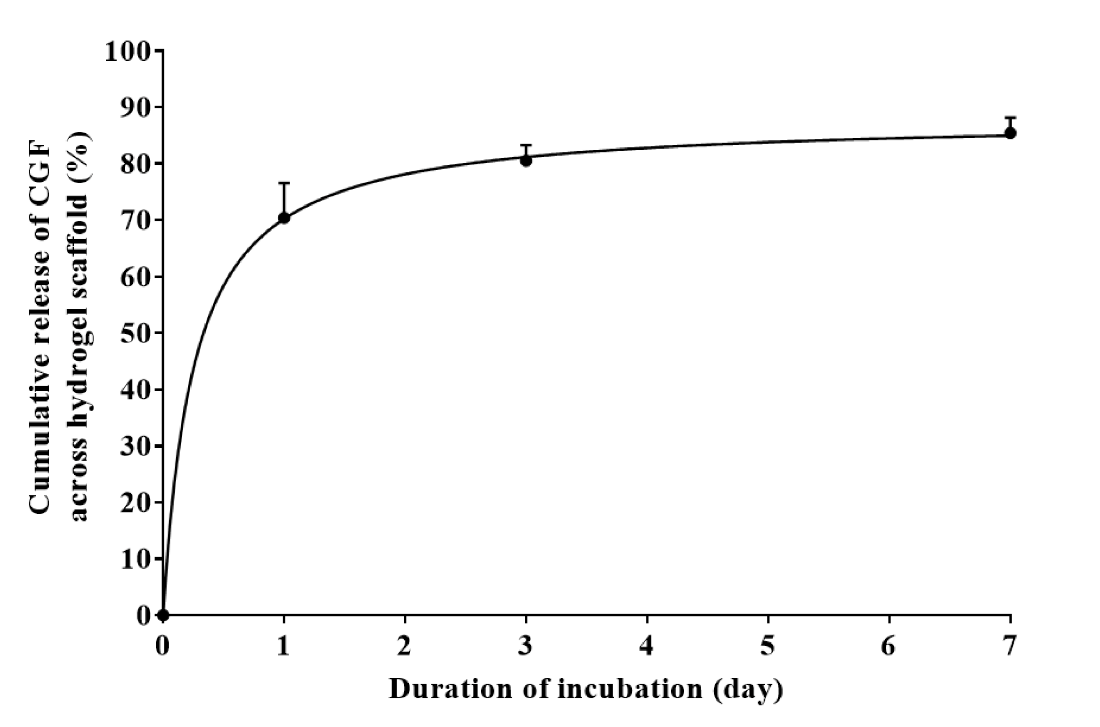
**Figure S5**. Cumulative percentage release of CGF across hydrogel scaffold examined with Franz diffusion cells. 800 µL of 1 g/L of CGF was incubated on top of 240 µL of hydrogel scaffold in the donor compartment. The donor and receptor compartments were separated by 2 sheets of 0.45 µm Whatman® filter papers. Samples of 1 mL were collected after 1, 3 and 7 d of incubation. The amount of CGF was determined based on UV-Vis spectroscopy. CGF in the donor compartment was replaced every 2 – 3 days (Mon, Wed, Fri). The mean (n = 3) ± SD are shown.

| **Table S1**. Identification of phenolic compounds in CGF and their negative mode ESI-Q-TOF MS data. | | | | |
| --- | --- | --- | --- | --- |
| **No.** | ***t*_R_ [min]** | **Area under curve (AUC)** | **Molecular formula** | **Calculated exact mass** |
| 1 | 1.6 | 547201 | Unidentified | 238.8918 |
| 2 | 2.0 | 2161837 | C_14_H_10_N_4_O_7_ | 346.0556 |
| 3 | 2.5 | 412131 | C_11_H_21_O_21_ | 489.0583 |
| 4 | 2.8 | 162878 | C_34_H_72_N_2_ | 508.5684 |
| 5 | 3.0 | 241069 | C_11_H_24_N_5_O_26_ | 642.0717 |
| 6 | 3.2 | 728555 | C_34_H_18_O_14_ | 650.0702 |
| 7 | 3.5 | 72264 | C_39_H_77_N_2_O_5_ | 653.5838 |
| 8 | 2.7 | 260400 | Unidentified | 484.5511 |
| 9 | 4.2 | 96054 | C_33_H_17_N_6_O_11_ | 673.0967 |
| 10 | 6.2 | 1312185 | Unidentified | 403.5568 |
| 11 | 7.0 | 1478331 | C_20_H_22_N_5_O_20_ | 652.0860 |
| 12 | 8.4 | 370695 | C_14_H_25_N_3_O_10_ | 395.1552 |
| 13 | 9.2 | 521467 | Unidentified | 491.6462 |
| 14 | 10.4 | 2075603 | C_36_H_32_O_20_ | 784.1493 |
| 15 | 10.9 | 500205 | C_21_H_17_N_3_O_9_ | 455.0971 |
| 16 | 11.3 | 116637 | C_17_H_29_O_11_ | 409.1712 |
| 17 | 12.6 | 499196 | C_24_H_41_O_16_ | 585.2404 |
| 18 | 13.0 | 210858 | C_18_H_31_O_11_ | 423.1873 |
| 19 | 13.8 | 1630126 | C_18_H_31_O_11_ | 423.1874 |
| 20 | 14.8 | 160296 | C_19_H_19_N_2_O_8_ | 403.1149 |
| 21 | 16.2 | 921071 | C_26_H_39_N_4_O_8_ | 535.2771 |
| 22 | 16.6 | 162573 | Unidentified | 685.8480 |
| 23 | 17.0 | 95147 | C_11_H_17_O_4_ | 213.1135 |
| 24 | 17.6 | 325275 | C_25_H_45_O_12_ | 537.2921 |
| 25 | 18.1 | 84804 | C_30_H_46_N_7_O_11_ | 680.3262 |
| 26 | 19.1 | 1512591 | C_31_H_35_NO_10_ | 581.2271 |
| 27 | 20.5 | 316501 | C_27_H_49_O_12_ | 565.3226 |
| 28 | 20.8 | 295379 | C_31_H_37_NO_10_ | 583.2428 |
| 29 | 22.5 | 834749 | C_33_H_39_NO_10_ | 609.2584 |
| 30 | 23.2 | 97231 | C_18_H_33_O_5_ | 329.2330 |
| 31 | 23.7 | 222649 | C_33_H_41_NO_10_ | 611.2744 |
| 32 | 27.0 | 454546 | C_17_H_25_O_4_ | 293.1761 |
| 33 | 27.5 | 2524827 | C_17_H_25_O_5_ | 309.1709 |
| 34 | 28.5 | 863702 | C_16_H_25_O_3_ | 265.1813 |
| 35 | 30.5 | 3071706 | C_17_H_25_O_3_ | 277.1810 |
| 36 | 33.8 | 3244893 | C_16_H_29_O_3_ | 269.2124 |
| 37 | 35.9 | 150481 | C_18_H_29_O_2_ | 277.2176 |
| 38 | 36.5 | 543737 | C_14_H_27_O_2_ | 227.2018 |
| 39 | 37.3 | 441208 | C_18_H_31_O_2_ | 279.2331 |
| 40 | 37.7 | 439026 | C_15_H_29_O_2_ | 241.2178 |
| 41 | 38.9 | 17801890 | C_16_H_31_O_2_ | 255.2331 |
| 42 | 40.0 | 1351697 | C_17_H_33_O_2_ | 269.2492 |
| 43 | 40.9 | 842536 | C_18_H_35_O_2_ | 283.2648 |
| 44 | 41.3 | 836042 | C_18_H_35_O_2_ | 283.2648 |

| **Table S2.** E_max_ & CC_50_ values of CGF (2D cell culture) | | | | | |
| --- | --- | --- | --- | --- | --- |
|  | **Concentration of CGF (g/L)** | | | | |
|  | **HDF** | **HaCaT** | **CHO** | **hMSC** | **ADSC** |
| E_max_ | 0.001 | 0.001 | 0.001 | 0.001 | 0.001 |
| CC_50_  (95% CI) | 0.597  (0.508  –  0.710) | 1.026  (0.957  –  1.139) | 0.755  (0.694  –  0.828) | 1.919  (1.486  –  2.881) | 4.645  (2.932  –  7.172) |

***Hydrodynamic size analysis of CGF via dynamic light scattering (DLS)***

Hydrodynamic size of the solubilized particles in CGF were first determined via DLS using Anton Paar Litesizer™ 500 (Graz, Austria), equipped with a red (658 nm) laser. The measuring range is between 0.3 nm – 10 µm in diameter, with a sensitivity of 0.1 mg/mL minimum macromolecule concentration. Briefly, 100 µL of CGF samples (n = 3) were examined in a low-volume quartz cuvette (163391, Anton Paar). Particle sizing was conducted in a series measurement mode at 25 °C. For each sample, 3 DLS measurements were conducted with 1 min of equilibration time in-between. The average hydrodynamic size of different populations of particles as well as their polydispersity index were analyzed with the software, Kalliope™ (Anton Paar).

| **Table S3.** Hydrodynamic size and polydispersity index of CGF. | | |
| --- | --- | --- |
| **Population** | **Average Hydrodynamic Size^†^ (nm)** | **Polydispersity Index (PDI)^‡^ (%)** |
| 1 | 920.4 ± 42.6 | 17.7 |
| 2 | 463.8 ± 15.3 | 30.9 |

^†^Hydrodynamic size was determined via DLS using Litesizer™ 500. The mean (n = 3) ± SD are shown.

^‡^PDI was determined via DLS using Litesizer™ 500. Both hydrodynamic size and PDI were examined at 25 °C.
